# Evaluation of Four Commercial Kits for SARS-CoV-2 Real-Time Reverse-Transcription Polymerase Chain Reaction Approved by Emergency-Use-Authorization in Korea

**DOI:** 10.3389/fmed.2020.00521

**Published:** 2020-08-13

**Authors:** Kyu-Hwa Hur, Kuenyoul Park, Youngkuen Lim, Yun Sil Jeong, Heungsup Sung, Mi-Na Kim

**Affiliations:** Department of Laboratory Medicine, Asan Medical Center, University of Ulsan College of Medicine, Seoul, South Korea

**Keywords:** severe acute respiratory syndrome coronavirus 2, coronavirus infectious disease 2019, emergency-use-authorization, real time, reverse transcription, polymerase chain reaction

## Abstract

SARS-CoV-2 real-time reverse-transcription PCR (rRT-PCR) is the most effective testing system currently available to counter COVID-19 epidemics when potent treatments and vaccines are unavailable. Therefore, four SARS-CoV-2 rRT-PCR kits have been approved by the emergency-use-authorization (EUA) without clinical validation in Korea until March 15, 2020. This study evaluated the analytical and clinical performance of these kits. Allplex 2019-nCoV Real-time PCR (Seegene, Seoul, Korea), PowerChek 2019-nCoV (KogeneBiotech, Seoul), Real-Q 2019-nCoV Real-Time Detection (BioSewoom, Seoul), and StandardM nCoV Detection (SD BIOSENSOR, Osong, Korea) were evaluated. The limit of detection (LODs) of Allplex, PowerChek, and Real-Q was determined by testing the transcribed RNA of SARS-CoV-2 *E* and the RNA of SARS-CoV Frankfurt1. A total of 27 consecutive samples comprising 13 sputum, 12 nasopharyngeal swab (NPS), 1 urine and 1 stool sample were collected from 2 COVID-19 patients for sensitivity analysis. Precision was assessed via daily tests of positive and negative controls in each kit for 5 d. Reproducibility was examined by repeating 21 samples and 10-fold dilutions of 14 samples in pairs using Allplex. Specificity was evaluated with 24 other respiratory virus-positive samples. LOD of Allplex, PowerChek, and Real-Q were 153.9, 84.1, and 80.6 copies/mL, respectively. The degrees of association between Cts and log viral concentrations by Allplex and PowerChek was expressed as y = −3.319 log (x) + 42.039 (R = 0.96) and y = −3.392 log(x) + 43.113 (R = 0.98), respectively. One or more of the 4 kits detected 20 out of 27 clinical samples positive. Of the 20 positive samples, the detection rates of positives for Allplex, PowerChek, Real-Q, and StandardM were 90.0, 82.3, 75.0, and 100.0%, respectively, but those of PowerChek and Real-Q would be 100% if out-of-cutoff Cts were counted as positives. Precision was 100%. Interpretation of Allplex results was reproducible when Ct of *E* ≤33. All 4 kits showed no cross-reactivity with other respiratory viruses. Performance of the 4 kits indicated the suitability of these for diagnosis and follow-up testing of COVID-19. Laboratory doctors who initially implement these EUA kits must be able to interpret quality control parameters.

## Introduction

Coronavirus disease 2019 (COVID-19) is an infectious disease caused by a novel coronavirus which was first detected in Wuhan city, China, in December 2019 (1–3). The virus was named severe acute respiratory syndrome coronavirus 2 (SARS-CoV-2). The World Health Organization (WHO) declared COVID-19 a pandemic on March 11, 2020 (4). Since the first SARS-CoV-2 case came to light, real time reverse transcription PCR (rRT-PCR) kits have been approved for use in Korea under Emergency-Use-Authorization (EUA). A total of 4 commercial SARS-CoV-2 rRT-PCR kits have been released to the market until 15 March 2020. As a result, 369,530 people were tested for SARS-CoV2 via rRT-PCR and 9,645 laboratory-confirmed COVID-19 cases were diagnosed nationwide until March 28 (21). The number of tests conducted was the highest in the world at the time and exceeded those conducted by China and Italy, the 2 countries with the highest number of COVID-19 patients. WHO emphasized the importance of laboratory diagnostics to control COVID-19 (5). Unlike China and Italy, rapidly expanding laboratory testing capabilities may have played an important role in mitigating the outbreak of COVID-19 in Korea (22). SARS-CoV-2 rRT-PCR is the most effective testing system available to counter epidemics when potent treatments and vaccines are unavailable. Therefore, commercial SARS-CoV-2 rRT-PCR kits have been approved by the EUA without clinical validation (6), because measures needed to counteract the COVID-19 pandemic require urgent deployment. However, all EUA kits should be prepared for clinical validation to obtain Ministry of Food and Drug Safety approval for the *in vitro* diagnostic kit before the end of the COVID-19 pandemic. It is, thereby, essential to evaluate their performance timely when the COVID-19 is less likely to end. The purpose of this study was to analytically and clinically validate 4 commercial kits approved by EUA in Korea.

## Methods

### Four SARS-CoV-2 Real-Time Reverse-Transcription PCR Commercial Kits

PowerChek 2019-nCoV Real-time PCR (PowerChek; KogeneBiotech, Seoul, Korea), Real-Q 2019-nCoV Detection (Real-Q; BioSewoom, Seoul, Korea), and StandardM nCoV Real-Time Detection (StandardM; SD BIOSENSOR, Osong, Korea) target regions of *envelope (E)* and *RNA dependent RNA polymerase* (*RdRp*) (1, 7), while Allplex 2019-nCoV (Allplex; Seegene, Seoul, Korea) has 3 targets, *E, RdRp* and *nucleocapsid protein (N)*. In accordance with WHO guidelines (8), the test is interpreted as positive when all target genes are detected together. If one target gene is positive, but the other is negative, the test is considered inconclusive. All kits performed reverse transcription and multiplex PCR in a single tube at one time, except PowerChek, which used 2 tubes per target gene. PowerChek and StandardM used exogenous internal controls in the rRT-PCR mixture, while Allplex used internal controls, spiking them directly into the sample prior to RNA extraction. Real-Q uses *human RNase P* intrinsic to human cells as an internal control. Specifications and PCR conditions of each kit are detailed (Table 1). This study was reviewed and approved by the Asan Medical Center Institutional Review Board and was deemed to be exempt from ethics and consent approval (2020-0487).

**Table 1:** Specifications and PCR conditions of the four commercial kits used for SARS-CoV-2 RNA detection.

**Kit name, manufacturer**	**PCR equipment**	**Target genes/No. tubes**	**Internal control**	**Volume of RNA eluates per test (μL)**	**No. amplification cycles**	**Running time of PCR**	**Cutoff Ct**
Allplex 2019-nCoV, Seegene	CFX96	*E, RdRp, N/*one	Bacteriophage MS2, spiked into sample	8	45	110 min	<40
PowerChek 2019-nCoV Real-time PCR, Kogene Biotech	CFX96, ABI7500, Gentier96	*E, RdRp*/two	Recombinant DNA plasmid spiked into PCR mixture	5	40	120 min	≤35
Real-Q 2019-nCoV Detection, BioSewoom	CFX96, ABI7500	*E, RdRp*/one	Human RNase P, intrinsic	5	40	110 min	Target gene: <38, Internal control: ≤35
StandardM nCoV Real-Time Detection, SD Biosensor	CFX96, ABI7500, LC480	*E, RdRp*/one	Pseudovirus particle, spiking of 5 μL into sample or 0.5 μL into PCR mixture	10	45, including 5 pre-amplifications	90 min	Target gene: ≤36^*^, Internal control: ≤32^*^

*SARS-CoV-2, severe acute respiratory syndrome coronavirus 2; Ct, cycle threshold*.

* *Pre-amplification was not counted for Ct*.

### Limits of Detection

The limit of detection (LOD) was determined using SARS coronavirus Frankfurt1 RNA (SARS-RNA) and *in vitro* transcribed RNA of *SARS-CoV-2 E* (*E-tRNA*) supplied by European Virus Archive-Global (https://www.european-virus-archive.com/) as published (7). *E-tRNAs* were serially diluted to obtain 5 concentrations as follows: 8, 80, 800, 8,000, and 80,000 copies/reaction for Allplex and 5, 50, 500, 5,000, and 50,000 copies/reaction for PowerChek, respectively. Twenty replicates of each sample were tested per concentration. Since Real-Q detected only SARS-RNAs of *E*, it was serially diluted to obtain 4 concentrations (5, 50, 500, and 5,000 copies/reaction), where each concentration was replicated 10 times. A response was not observed for both RNAs in *E* PCR using StandardM. LOD was calculated via probit regression analysis using MedCalc (version 19.2; MedCalc Software, Ostend, Belgium). Correlation between each concentration and cycle threshold (Ct) was calibrated via linear regression to calculate Ct matching the LOD using MedCalc.

### Evaluation of Sensitivity and Specificity With Clinical Samples

**Figure 1:**
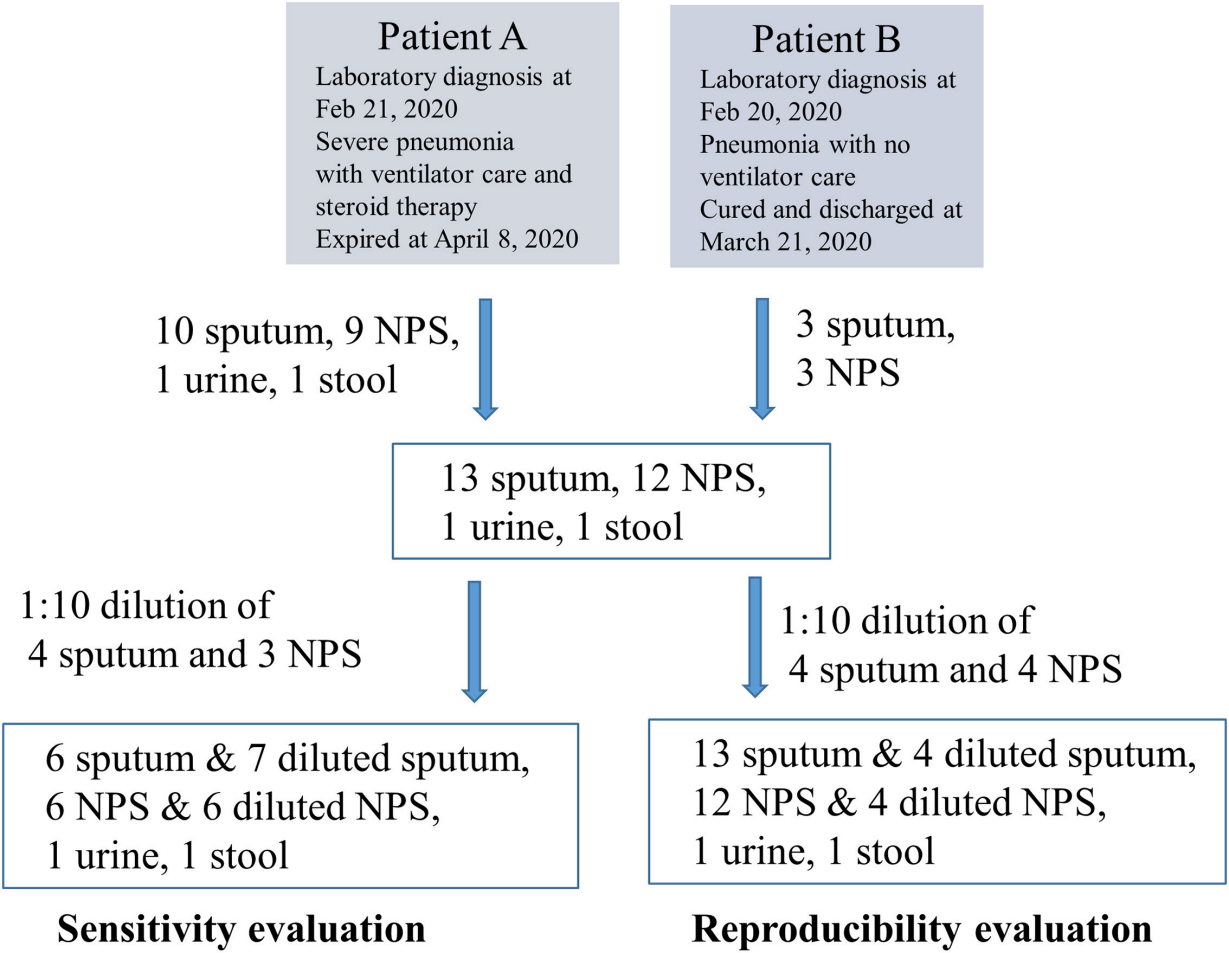
Summary of the clinical specimens for evaluating sensitivity and reproducibility.

A total of 27 samples were collected from 2 patients between February 29 and March 10 as follows: 10 sputum, 9 nasopharyngeal swab (NPS), 1 urine, and 1 stool sample consecutively from one COVID-19 patient with prolonged viral shedding (A) (9); and 3 sputum and 3 NPS samples consecutively from the other (B) (Figure 1). RNA was extracted using eMAG (bioMérieux, Marcy-l'Etoile, France) to the extent of 0.05 mL of eluent per 0.2 mL sample and routinely tested via an Allplex 2019-nCoV (Seegene) using a Bio-Rad CFX96 instrument (Bio-Rad, Hercules, CA). Remaining samples were stored at −70°C, thawed once, and used on the same day to test the 4 commercial kits. A total of 12 samples, including 7 of the 13 sputum samples and 5 of the 12 NPS samples, were diluted 10-fold to enrich weak positive samples. The other 3 kits were tested using an ABI7500 instrument (Applied Biosystems, Foster City, CA). Interpretation of results and Ct values of the target genes and internal controls of the 4 kits were compared. The test results of each kit were interpreted following the manufacturer's recommendations as follows: positive only if both the Ct value of the target gene and the internal control were within the cutoff. If a sample was positive for 1 or more kits, it was considered truly positive for sensitivity purposes, because it was a serial sample obtained from a laboratory-confirmed COVID-19 case. Two NPS samples and 1 sputum sample were not subjected to PowerChek testing, due to insufficient sample volume.

In order to assess the specificity of each kit, 24 respiratory virus-positive samples including 21 NPS samples, and 3 sputum samples were tested for cross-reactivity with human RNA or other respiratory viruses. Samples were determined as positive for a respiratory virus using an Allplex Respiratory Panel 1/2/3 (Seegene). The 24 samples consisted of the human rhinovirus (*n* = 9), respiratory syncytial virus B (*n* = 6), human adenovirus (*n* = 3), human coronavirus 229E (*n* = 2), human coronavirus OC43 (*n* = 1), human coronavirus NL63 (*n* = 1), influenza virus A (*n* = 1), and human metapneumovirus (*n* = 1).

The Ct values of the internal control were analyzed for PCR inhibition effects by sample type.

### Precision Tests

One positive control and one negative control in each kit were repeatedly tested once a day for 5 d. The results were analyzed and interpreted as positive or negative and the Ct value of each target gene for positive control. Comparison of Ct value and their coefficient of variance were performed using MedCalc.

To evaluate the reproducibility of the clinical sample test, using Allplex, 21 samples were freeze-thawed once and tested a second time in a single run, while 14 samples were tested in pairs with 10-fold-diluted samples. Evaluation of the reproducibility of the repeat test was based on the consistency of positive and negative results. Delta Ct values between the initial and repeated tests of the same sample and between original and 10-fold-diluted samples were also calculated.

## Results

**Figure 2:**
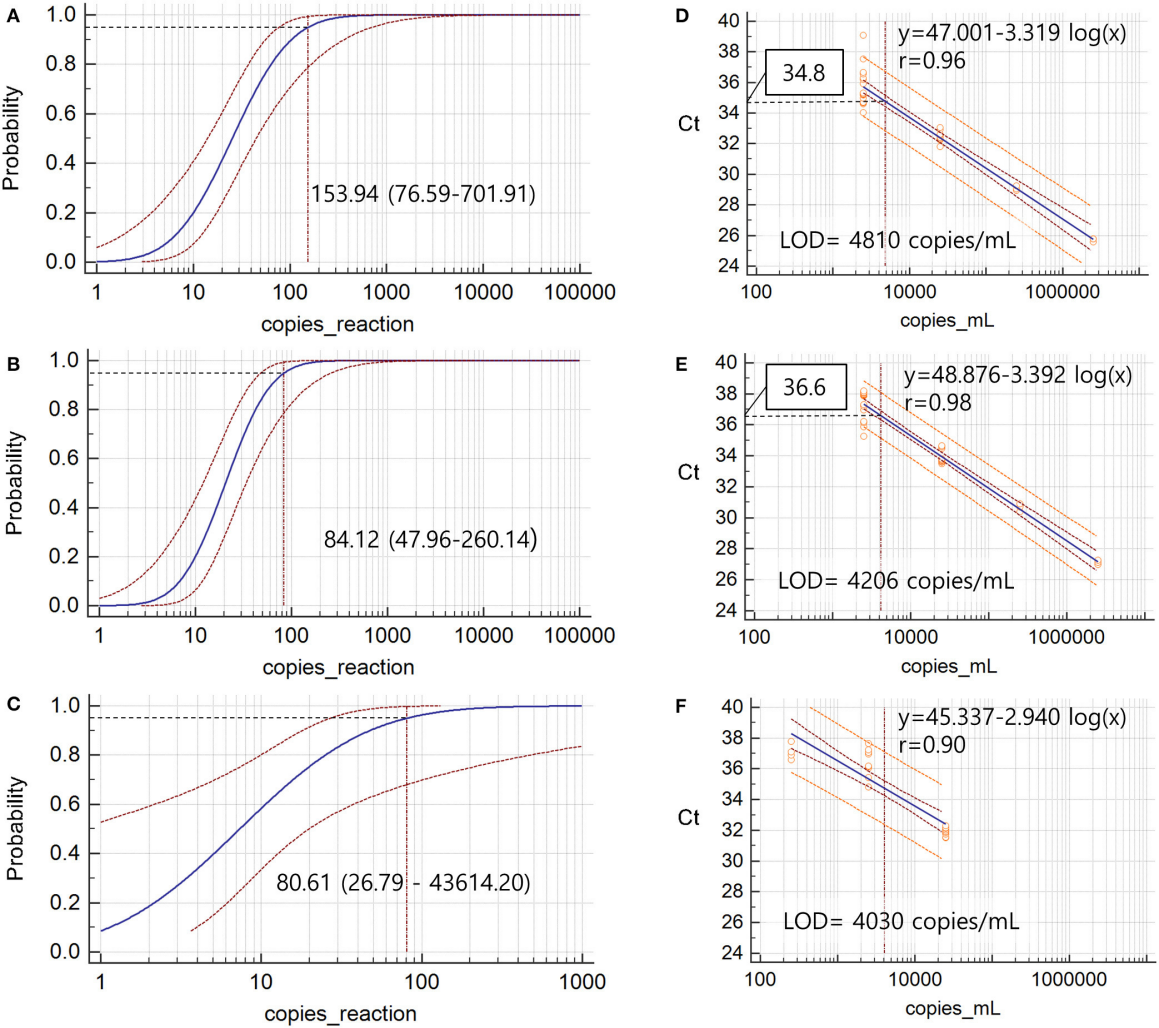
The limits of detection of 95% (LOD) via probit analysis based on 20 replicates of serially diluted *in vitro* transcribed *E* RNA from SARS-CoV-2 for Allplex **(A)** and PowerChek **(B)** and 10 replicates of serially diluted purified RNA from SARS coronavirus Frankfurt1 for Real-Q **(C)**. Black dotted lines indicated LOD and values are denoted with 95% confidence intervals in parenthesis. The correlation between Cts and viral loads was analyzed via linear regression based on LOD experiments in Allplex **(D)**, PowerChek **(E)**, and Real-Q **(F)**. Open orange circles represent the results of each test. The Ct values corresponding to LOD of Allplex and PowerChek were calculated using the linear regression equation and are denoted by boxes.

LOD values, the calculation of which were based on the power of detecting *E-tRNA*, were 153.94 (95% CI 76.59–701.91) copies/reaction for Allplex and 84.12 copies/reaction (95% CI 47.96–260.14) for PowerChek, respectively. LOD value for Real-Q, the calculation of which was based on the power of detecting *SARS-RNA* was 80.60 (95% CI 26.78–43,614.20) copies/reaction, respectively (Figure 2). Detection sensitivity per sample volume determined via Allplex, PowerChek, and Real-Q were 4.81 × 10^3^/mL, 4.21 × 10^3^/mL, and 4.03 × 10^3^/mL, respectively.

At the concentration ranges defined for LOD experiments, the degrees of association between Ct values and log viral concentrations, indicated by Allplex and PowerChek, were expressed by the log linear equations, y = −3.319 log (x) + 42.039 (R = 0.96) and y = −3.392 log(x) + 43.113 (R = 0.98), respectively (Figure 1). The average Ct values of Real-Q at 5, 50, and 500 copies per reaction were 37.1 ± 0.51, 36.4 ± 0.91, and 31.9 ± 0.26, respectively, and, as shown (Figure 1), there was a lack of linear relationship (y = −2.940x + 40.341 within this range (R = 0.90). Ct values corresponding to LOD that were estimated using these equations were 34.8 for Allplex and 36.6 for PowerChek.

Initial interpretation of the Allplex test results indicated that the 27 clinical samples consisted of 19 positive, 6 inconclusive, and 2 negative samples. Allplex readings showed that the 20 *E*-positive samples comprised 7, 4, 5, and 4 samples with *E* Ct values larger than 33 (E33), 30–33, 25–30, and <25, respectively, and 7 *E*-negative samples (E0) (Table 2). Ct readings for both target genes provided by PowerChek or Real-Q were found to lie outside the cutoff values for one or more target genes, in 4 and 5 samples, respectively. Allplex. PowerChek, Real-Q, and StandardM detected 18, 14, 15, and 20 positive samples and 4, 1, 6 and 5 inconclusive samples, respectively. One stool sample and 1 urine sample were positive for only *RdRp* via StandardM and only 1 *N* by Allplex, respectively, and were therefore excluded from sensitivity calculations. Thus, according to the combined results of all kits, a total of 25 samples comprising 20 positives and 5 inconclusive ones were obtained and used to calculate sensitivity. All 4 kits were 100% sensitive for *E* Ct value ≤33, regardless of sample type. By contrast, 4 E33 and 1 E0 positive and 4 E0 and 1 E33 inconclusive samples showed discrepancies between the 4 kits as follows: 2 positives to inconclusive and 4 inconclusive ones to negative in Allplex; 1 E33 positives to inconclusive and 2 positives and all 5 inconclusive ones to negative in PowerChek; and all 4 positives to inconclusive and 3 inconclusive ones to negative in Real-Q. StandardM correctly detected all positive and one inconclusive to negative. Therefore, the sensitivity for detecting positive samples in Allplex, PowerChek, Real-Q, and StandardM was 90.0% (18/20), 82.3% (14/17), 75.0% (15/20), and 100.0% (20/20), respectively. Because out-of-cutoff Ct values were present only in PowerChek and Real-Q, their sensitivities were increased to 100%, if the samples were considered positive when both target genes were detected via out-of-cutoff Ct values. When analyzing consecutive samples obtained from patient A, all inconclusive results were accompanied by other samples that were positive, or followed by samples that became positive the next day.

**Table 2:** Evaluation of sensitivity of the four commercial kits with consecutive samples from two COVID-19 patients.

**Specimen No**.	**Type of specimen**	**Results and Ct values if it is positive**																
		**Allplex**					**PowerChek**				**Real-Q**				**StandardM**			
			***E***	***RdRp***	***N***	**IC**		***E***	***RdRp***	**IC**		***E***	***RdRp***	**IC**		***E***	***RdRp***	**IC**
A0229st	Stool	N	–	–	–	26.1	N	–	–	19.4/18.9	N	–	–	–	I	–	32.2	19.3
A0229ur	Urine	I	–	–	39.3	29.6	N	–	–	19.5/18.9	N	–	–	34.2	N	–	–	19.1
A0229np	NPS	P	32.1	33.7	35.2	30.2		NA	NA	NA/NA	P	34.3	33.7	27.5	P	28.4	27.0	19.6
A0301np1:10	NPS	P	28.7	30.8	32.5	30.9	P	29.1	29.5	18.7/18.2	P	30.9	30.2	31.3	P	24.0	23.7	19.5
A0303np	NPS	P	34.6	36.6	36.1	27.6		NA	NA	NA/NA	I	38.4	36.4	28.2	P	28.7	28.7	19.2
A0304np1:10	NPS	P	28.9	31.0	32.1	30.8	P	30.0	30.2	18.8/18.3	P	31.2	30.6	29.3	P	24.5	24.4	19.5
A0305np	NPS	N	–	–	–	25.6	N	–	–	18.7/18.2	I	–	37.1	29.3	N	–	–	19.3
A0306np	NPS	N	–	–	–	26.2	N	–	38.2	18.6/18.3	N	–	39.0	25.8	I	–	32.1	19.3
A0307np	NPS	I	33.9	–	37.0	24.6	N	35.1	37.3	18.6/18.4	I	38.3	37.2	28.0	I	30.4	–	19.5
A0309np	NPS	N	–	–	–	25.1	N	35.1	–	18.6/18.5	N	–	38.3	26.3	I	–	30.9	19.7
A0310np	NPS	I	–	–	37.3	25.2	N	37.0	37.0	18.7/18.4	I	38.9	37.8	25.2	P	31.3	30.3	19.2
A0229sp1:10	Sputum	P	19.9	21.8	23.8	–	P	21.4	21.6	18.7/18.2	P	22.5	21.9	28.9	P	15.8	15.4	19.0
A0301sp1:10	Sputum	P	27.8	29.7	31.1	30.5	P	28.8	28.0	18.7/18.1	P	30.2	29.7	28.9	P	23.3	23.0	19.5
A0303sp1:10	Sputum	P	30.3	32.9	32.6	32.3	P	31.8	32.1	18.8/18.3	P	32.9	31.9	28.0	P	25.2	25.5	19.3
A0304sp1:10	Sputum	P	33.2	34.1	35.0	33.9	P	33.6	34.1	18.7/18.3	P	36.1	35.7	27.9	P	28.4	28.6	19.5
A0305sp	Sputum	P	31.2	34.3	34.6	30.0	P	33.5	34.8	18.7/18.2	P	35.0	34.6	24.1	P	28.2	27.8	19.3
A0306sp	Sputum	P	33.5	36.2	35.5	27.3	I	34.7	35.2	18.6/18.3	I	38.3	35.3	24.4	P	28.9	27.9	19.2
A0307sp	Sputum	P	34.3	35.0	36.9	28.0	N	36.1	37.6	18.7/18.0	I	38.4	37.1	24.0	P	30.2	29.1	19.2
A0308sp	Sputum	I	33.5	–	39.5	32.1		NA	NA	NA/NA	N	39.6	38.2	23.0	P	29.2	30.2	19.8
A0309sp	Sputum	P	34.4	38.7	36.9	29.0	P	34.3	34.9	18.7/18.5	P	36.8	34.9	22.2	P	28.9	29.9	19.6
A0310sp	Sputum	N	–	–	–	25.1	N	–	–	18.7/18.4	N	–	–	24.4	I	31.3	–	19.2
B0308np1:10	NPS	P	24.1	25.9	27.0	28.1	P	24.5	25.4	18.8/18.2	P	26.2	26.1	29.9	P	19.5	19.3	19.3
B0309np1:10	NPS	P	22.7	24.9	26.1	25.9	P	23.3	24.8	18.6/18.2	P	25.1	24.7	29.1	P	18.5	17.9	19.2
B0310np1:10	NPS	P	26.6	29.0	29.5	28.6	P	27.0	28.0	18.6/18.1	P	28.8	28.3	29.4	P	22.3	21.8	19.3
B0308sp1:10	Sputum	P	21.1	23.0	23.7	–	P	22.4	22.6	18.6/18.1	P	23.6	23.4	28.6	P	17.3	16.8	19.0
B0309sp1:10	Sputum	P	28.0	30.3	31.2	28.6	P	28.3	28.8	18.6/18.1	P	29.9	29.1	31.7	P	23.1	22.8	19.3
B0310sp1:10	Sputum	P	31.0	32.9	34.6	28.9	P	32.6	33.8	18.5/18.1	P	32.6	31.7	31.6	P	25.5	25.5	19.2

*Specimen number is a combination of patient (A or B), specimen date (MMDD), type of specimen (st, stool; ur, urine; np, nasopharyngeal; sp, sputum), and 1:10 if diluted samples were used for the evaluation. Two Ct values for the internal control were produced from each reaction for E and RdRp, respectively, in the PowerChek column. Shaded Ct value were in out of cutoff range; >35 for PowerChek and >38 for Real-Q*.

*Ct, cycle threshold; IC, internal control; P, positive; N, negative; I, inconclusive; NPS, nasopharyngeal swab; NA, not available*.

Twenty four samples, that tested positive for other respiratory viruses, tested negative in all 4 SARS-CoV-2 rRT-PCR kits. Therefore, all kits showed 100% specificity. Allplex, PowerChek, and Real-Q showed a cross-reaction to *E* of SARS-CoV Frankfurt1 while StandardM was negative for both *E* and *RdRp*, when tested with SARS-RNA.

Since all positive and negative controls consistently showed expected results, indicating that the precision of qualitative tests was 100% (Table 3). Mean Ct values for *E* of the positive controls in Allplex, PowerChek, Real-Q, and StandardM were 20.0 ± 0.23, 23.1 ± 0.16, 27.2 ±.13, and 26.2 ± 0.22, respectively. Among the 4 kits, the smallest coefficient of variation of Ct values was observed for Real-Q. *RdRp* showed wider variation than other target genes in PowerChek (Table 3). When Ct values were compared between *E* and *RdRp* within the kits, there was no significant difference in Allplex and PowerChek, while *RdRp* was consistently longer by 1.54 in average than *E* in Real-Q and vice versa by 0.72 in StandardM. *N* showed the shortest Ct in Allplex (Table 3). In the reproducibility test, conducted using clinical samples, 8 samples, including 1 stool, 3 NPS, and 4 sputum samples, all of which were E33 or negative for *E*, displayed discrepancies in interpretation of results. Thirteen pairs of samples were available for the comparison of Ct values of *E*, and delta Ct values were 1.0 or less, except for 2 samples, with Cts of 32.4 and 34.4, respectively. Thus, if the Ct was not longer than 33, both the interpretation and Ct values of the target gene derived via Allplex were consistently reproducible. When 14 pairs of original (undiluted) and 10-fold-diluted samples were tested, one sample with a Ct for *E* of 34.6 was converted from positive to inconclusive, after dilution. For the remaining 13 pairs of original samples with Ct values for *E* ranging from 17.6 to 32.5, interpretation was consistently positive and when tested via 10-fold-dilutions, the average delta Ct for these pairs was 3.7 ± 1.2 (Table 4).

**Table 3:** Precision tests for four commercial kits.

**Run No**.	**Ct values of target genes**												
	**Allplex**				**PowerChek**			**Real-Q**			**StandardM**		
	**Positive control**			**Negative control**	**Positive control**		**Negative control**	**Positive control**		**Negative control**	**Positive control**		**Negative control**
	***E***	***RdRp***	***N***		***E***	***RdRp***		***E***	***RdRp***		***E***	***RdRp***	
Run 1	19.9	20.7	19.2	ND	22.9	23.8	ND	27.0	27.6	ND	26.1	24.3	ND
Run 2	20.4	20.3	19.5	ND	23.3	22.5	ND	27.1	27.8	ND	26.4	25.1	ND
Run 3	20.0	20.5	19.3	ND	23.0	22.3	ND	27.3	27.8	ND	26.1	24.8	ND
Run 4	19.8	20.0	18.5	ND	23.2	22.7	ND	27.2	27.9	ND	26.4	24.7	ND
Run 5	19.9	20.1	18.5	ND	23.0	21.7	ND	27.3	27.9	ND	25.9	24.3	ND
Mean	20.0	20.3^a^	19.0^b^	ND	23.1	22.6^a^	ND	27.2	27.8^a^	ND	26.2	24.6^a^	ND
SD	0.23	0.29	0.47		0.16	0.77		0.13	0.12		0.22	0.34	
CV (%)	1.17	1.41	2.47^c^		0.71	3.4^c^		0.48	0.44^c^		0.83	1.39^c^	

*ND, not detected; SD, standard deviation; CV, coefficient of variation*,

a *Comparison of Ct using the Mann Whitney U-test between E and RdRp showed significant difference in Real-Q (p = 0.008) and StandardM (p = 0.008)*,

b *Ct of N was shorter than E and RdRp in comparison using Kruskall Wallis test of three target genes of Allplex (p = 0.004)*,

c *Comparison of the CV using F-test between target genes within the kits showed that RdRp is bigger than E in PowerChek (p = 0.005)*.

**Table 4:** Reproducibility test using Allplex 2019-nCoV Assay.

**Specimen No**.	**Type of specimen**	**Ct values of target genes and internal control**											
		**1st test**				**2nd test**				**Test with 1:10 dilution**			
			***E***	***RdRp***	***N***		***E***	***RdRp***	***N***		***E***	***RdRp***	***N***
A0229st	Stool	I	36.8	–	–	N	–	–	–				
A0229ur	Urine	I	–	39.8	–	I	–	–	39.3				
A0229np	NPS	P	32.5	34.1	35.2	P	32.1	33.7	35.2	P	35.0	35.7	38.2
A0301np	NPS	P	25.4	26.3	28.8	P	25.2	26.7	28.9	P	28.7	30.8	32.5
A0303np	NPS	P	34.6	36.6	36.1	P	33.0	34.0	36.2	I	–	–	37.6
A0304np	NPS	P	25.3	27.3	28.8	P	25.7	27.1	28.8	P	28.9	31.0	32.1
A0305np	NPS	P	35.2	37.2	35.8	N	–	–	–				
A0306np	NPS	N	–	–	–	N	–	–	–				
A0307np	NPS	I	33.0	–	37.7	I	33.9	–	37.0				
A0309np	NPS	N	–	–	–	N	–	–	–				
A0310np	NPS	P	35.8	38.2	38.2	I	–	–	37.3				
A0229sp	Sputum	P	16.1	17.2	19.0	P	16.4	18.0	20.2	P	19.9	21.8	23.8
A0301sp	Sputum	P	24.7	28.7	27.6	P	24.4	26.0	27.6	P	27.8	29.7	31.1
A0303sp	Sputum	P	26.1	29.5	29.4	P	27.1	29.1	29.5	P	30.3	32.9	32.6
A0304sp	Sputum	P	29.8	31.4	31.4	P	30.1	31.6	31.9	P	33.2	34.1	34.9
A0305sp	Sputum	P	32.4	34.3	35.0	P	31.2	34.3	34.6				
A0306sp	Sputum	P	33.6	34.6	35.7	P	33.5	36.2	35.5				
A0307sp	Sputum	I	–	36.8	–	P	34.3	35.0	36.9				
A0308sp	Sputum	P	33.4	36.8	39.8	I	33.5	–	39.5				
A0309sp	Sputum	I	36.0	–	38.6	P	34.4	38.7	36.9				
A0310sp	Sputum	I	37.1	–	–	N	–	–	–				
B0308np	NPS	P	19.9	22.0	23.0					P	24.1	25.9	27.0
B0309np	NPS	P	19.1	24.7	23.8					P	22.7	24.9	26.1
B0310np	NPS	P	22.6	24.5	26.8					P	26.6	29.0	29.5
B0308sp	Sputum	P	17.6	19.4	20.1					P	21.1	23.0	23.7
B0309sp	Sputum	P	23.6	25.6	27.3					P	28.0	30.3	31.2
B0310sp	Sputum	P	26.0	28.0	30.0					P	31.0	32.9	34.6

Internal control (IC) was exogenous in 3 kits and endogenous in Real-Q (Table 1). In sensitivity assessment testing, the average Ct values of the ICs were 29.3 ± 3.9 for Allplex, 18.3 ± 0.2 for PowerChek, 27.7 ± 2.9 for Real-Q, and 19.3 ± 0.2 for StandardM (Table 2). The Cts for IC were missed in 2 sputum samples, when tested using Allplex, and in 1 stool sample, when tested using Real-Q. In addition, one urine sample showed the longest Ct value of 34.2 for ICs when tested via Real-Q. When comparing the 7 pairs of sputum and NPS tested simultaneously via Allplex, Ct values for ICs were consistently longer for sputum samples than those for NPS (28.8 ± 2.7 vs. 26.2 ± 2.1), although those of the other kits were not (Table 2). Real-Q and StandardM indicated that the Cts for ICs were <35 and <32, respectively, and that all Ct values for the ICs of the NPS and sputum samples tested were <32 and <20, respectively.

## Discussion

The 95% confidence intervals estimated for Allplex, PowerChek and Real-Q overlapped, indicating that there was no significant difference in LODs between test kits. However, the LODs for those kits were more than 10-fold less sensitive compared to the previously reported 5.2 copies/reaction (7). Nevertheless, this LOD was consistent with the 295 copies/reaction stated by another study that validated a diagnostic kit using the Cobas 6800 (10). The two previous studies used standard materials and methods in accordance with WHO guideline, and were therefore similar to those used in this study (7, 8, 10). Compared with that the LOD of the commercial kits assayed using culture viral RNA varied from 3.3 to 330 copies/reaction in the evaluation of 11 commercial kits including PowerChek (11), all three kits had similar sensitivity and LODs in viral concentrations were alsp in the range of 4.03 × 10^3^ to 4.81 × 10^3^ copies/mL of samples. Log linearity between the Cts and viral loads showed that the best fit slopes of Allplex and PowerChek was closed to the ideal value of −3.3219 (12). PCR efficiency of Allplex has recently been published similarly high as 105% for E gene (13). This high PCR efficiency was suggesting the possibility of quantifying SARS-CoV-2 at concentrations higher than the LOD.

Although a corresponding LOD was not measured, StandardM exhibited the highest clinical sensitivity among the 4 kits. This difference in sensitivity was due to intentional enrichment of samples with very low viral loads, where all kits showed 100% reproducible results for samples with a Ct ≤33. In the recent evaluation with low viral concentration samples with Ct >34.5 in E gene of in-house PCR, Allplex sensitively detected those as 12 out of 13 (13). Therefore, all kits were sufficiently sensitive for initial diagnostic testing. In addition, there was no risk of misinterpreting positive to truly negative readings, even when one or more multiple target genes were either positive or out-of-cutoff positive. Considered together, these findings point to excellent PCR efficiency which also enabled quantitative measurements, and thus these tests may be used to monitor viral loads in follow-up samples. The Korea Center for Disease Control and Prevention (KCDC) stipulated that the criterion for releasing COVID-19 patients from quarantine isolation was receipt of 2 consecutive negative test results at 24 h-intervals (14). Currently, studies on SARS-CoV-2 viral loads that are sufficiently infectious to require quarantine isolation are scant. By May 15, 2020, 9,821 confirmed cases had been released from quarantine, in accordance with KCDC criteria, and reportedly, no new infections had been transmitted by those who were released, although retesting showed that a total of 447 of those released had turned positive later (15). This finding supported the use of rRT-PCR kits upon the EUA for follow-up tests of COVID-19. This may be possible because Korean COVID-19 laboratory response task force defines the quality parameters of COVID-19 diagnostic kits and validate the performance of all EUA kits (6, 16). This EUA model originates from the 2015 MERS outbreak laboratory response task force (17, 18), which has the added advantage of providing reliable COVID-19 diagnostic kits nationwide.

All 4 kits did not show positivity to respiratory viruses other than SARS-CoV-2. Most importantly, none of these 4 commercial kits exhibited cross reactions with other human coronaviruses including OC43, 229E, NL63, and *RdRp* of SARS-CoV Frankfurt1. Because the kits tested in this study were all based on WHO recommendations, specificity appeared to be consistent with that reported by previous studies (7, 10). However, there was no MERS coronavirus included in the specificity panel and there was the report that E gene of PowerChek cross-reacted with MERS coronavirus (11). Because no MERS is endemic in Korea, cross reactivity with MERS coronavirus is not significant limitation for SARS-CoV-2 diagnostic kits.

The precision of these 4 kits for qualitative testing was 100% in 5 replicates of positive and negative controls. Although standard assessments require 10 replicates, testing positive and negative controls twice a day for 5 days, precision could be more confident by that Cts of target genes for five replicates were also highly consistent for each gene and comparable between the genes in the kits. Reproducibility of clinical test results was excellent for Cts ≤33. This reproducibility may be due to the high-efficacy of PCR which ensured excellent precision (12). PowerChek exhibited the most negative results, 7, with 1 inconclusive, showing the beneficial effect of reduced uncertainty, which however, may need further study. This finding is associated with PowerChek setting the cutoff higher than the LOD, while Allplex set it lower than the LOD. These cutoff settings raise a query as to whether negative or inconclusive results, obtained by including out-of-cutoff positives for one of the multiple target genes, are truly negative and indicate lack of infectiousness. In the current study, follow-up samples of laboratory-confirmed COVID-19 patients often showed inconclusive results. However, these should not be interpreted as true negative, as they may be accompanied by or followed by other positive results, and thus may be in the very early or late stages of COVID-19. If the sample showing out-of-cutoff positivity is the first from a new patient, SARS-CoV-2 rRT-PCR should be retested with a resample to confirm COVID-19. One of the advantages of diagnostic kits based on multiple target genes is that results can be interpreted as those of a combination of target genes, which complements both sensitivity and specificity.

A major challenge encountered in standard real-time PCR analysis is the elimination of false negative signals, caused by inhibitors or inefficient PCR conditions (12, 19). Internal controls are used to address reliability, via the addition of extra primer-probe sets targeted to other endogenous DNA sequences or exogenous targets (20). The Cts of ICs in sputum samples was consistently longer than that of NPS when tested by Allplex, where these sputum samples were subjected to more inefficient PCR conditions. This finding was consistent with that of a previous study (18). While PowerChek and StandardM showed invariable Cts for ICs, regardless of individual samples or sample types, the ICs of Allplex were more sensitive indicators of PCR efficiency than those of other kits. The ICs of Real-Q showed longer Cts or even failed to amplify, as seen in urine and stool samples, suggesting that the intrinsic IC system of Real-Q may not be compatible with samples other than respiratory samples. In pandemic situations, such as that of COVID-19, quality control of ICs is essential, especially for introducing an EUA-approved rRT-PCR kit to the diagnostic equipment market.

This study was beset with some limitations. Firstly, analytical sensitivity of all 4 kits and all target genes was not validated due to lack of appropriate standards. Although only the LOD of *E* was analyzed, other target genes also showed comparable Cts in the precision analysis of positive control and equivalent sensitivity in clinical sample analyses. It is important to establish reliable standards for further validation of EUA-approved kits. Secondly, all clinical samples were consecutively collected from 2 patients, and thus did not represent clinical samples from initial diagnoses. This study was intended to enrich low viral load samples with a Ct close to a value to that of a follow-up test, especially for interpretation of results when multiple target genes were used in combination. Thirdly, the range of negative samples used was relatively small and failed to include all common respiratory pathogens and all human coronaviruses in specificity testing.

In conclusion, the precision and accuracy of the 4 commercial kits indicated that the properties expected of an effective diagnostic device have been met. IC system of Allplex was the most sensitive in monitoring PCR efficiency. High efficiency of PCR and log linear relationship between Ct and viral load in Allplex and PowerChek suggested the use for quantification of viral load and follow-up testing of COVID-19 as an indicator of quarantine release. Therefore, all four kits can be used to diagnose and follow up COVID-19 for treatment and discharge planning, as well as to estimate viral load if there is a reliable standard. It is expected that the laboratory doctors will possess a good understanding of the quality control parameters needed for interpreting results and troubleshooting issues, when using EUA kits in clinical laboratory settings.

## Data Availability Statement

The original contributions presented in the study are included in the article, further inquiries can be directed to the corresponding author.

## Ethics Statement

The studies involving human participants were reviewed and approved by Asan Medical Center Institutional Review Board. Written informed consent for participation was not required for this study in accordance with the national legislation and the institutional requirements.

## Author Contributions

M-NK led the entire study from ideation to writing this paper. HS collaborated in all of M-NK's work. YL and YJ conducted the experiments. K-HH and KP analyzed the data and wrote this paper. All authors contributed to the article and approved the submitted version.

## Conflict of Interest

The authors declare that the research was conducted in the absence of any commercial or financial relationships that could be construed as a potential conflict of interest.
